# Semaglutide Improves Liver Steatosis and De Novo Lipogenesis Markers in Obese and Type-2-Diabetic Mice with Metabolic-Dysfunction-Associated Steatotic Liver Disease

**DOI:** 10.3390/ijms25052961

**Published:** 2024-03-04

**Authors:** Manuel Soto-Catalán, Lucas Opazo-Ríos, Hernán Quiceno, Iolanda Lázaro, Juan Antonio Moreno, Carmen Gómez-Guerrero, Jesús Egido, Sebastian Mas-Fontao

**Affiliations:** 1 Renal, Vascular and Diabetes Research Laboratory, IIS-Fundación Jiménez Díaz, Spanish Biomedical Universidad Autónoma de Madrid, 28049 Madrid, Spain; manuel.sotoc@quironsalud.es (M.S.-C.); lopazo@udla.cl (L.O.-R.); cgomez@fjd.es (C.G.-G.); 2 Research Centre in Diabetes and Associated Metabolic Disorders (CIBERDEM), 28029 Madrid, Spain; 3 Facultad de Ciencias de la Salud, Universidad de Las Américas, Concepción-Talcahuano 4301099, Chile; 4 Department of Pathology, Fundación Jiménez Díaz, 28040 Madrid, Spain; hernan.quiceno@fjd.es; 5 Cardiovascular Risk and Nutrition Research Group, Epidemiology and Public Health Program, Hospital del Mar Medical Research Institute (IMIM), 08003 Barcelona, Spain; iolan.lazaro@researchmar.net; 6 Department of Cell Biology, Physiology and Immunology, University of Cordoba, 140471 Cordoba, Spain; juan.moreno@uco.es; 7 Maimónides Biomedical Research Institute of Cordoba (IMIBIC), Hospital Universitario Reina Sofía, 14004 Córdoba, Spain; 8 Faculty of Medicine and Biomedicine, Universidad Alfonso X el Sabio (UAX), 28691 Madrid, Spain

**Keywords:** diabetes, insulin resistance, GLP1 receptor agonists, obesity, semaglutide, steatosis

## Abstract

Metabolic-dysfunction-associated steatotic liver disease (MASLD) is a prevalent clinical condition associated with elevated morbidity and mortality rates. Patients with MASLD treated with semaglutide, a glucagon-like peptide-1 receptor agonist, demonstrate improvement in terms of liver damage. However, the mechanisms underlaying this beneficial effect are not yet fully elucidated. We investigated the efficacy of semaglutide in halting MASLD progression using a genetic mouse model of diabesity. Leptin-receptor-deficient mice with obesity and diabetes (BKS db/db) were either untreated or administered with semaglutide for 11 weeks. Changes in food and water intake, body weight and glycemia were monitored throughout the study. Body fat composition was assessed by dual-energy X-ray absorptiometry. Upon sacrifice, serum biochemical parameters, liver morphology, lipidomic profile and liver-lipid-related pathways were evaluated. The semaglutide-treated mice exhibited lower levels of glycemia, body weight, serum markers of liver dysfunction and total and percentage of fat mass compared to untreated db/db mice without a significant reduction in food intake. Histologically, semaglutide reduced hepatic steatosis, hepatocellular ballooning and intrahepatic triglycerides. Furthermore, the treatment ameliorated the hepatic expression of de novo lipogenesis markers and modified lipid composition by increasing the amount of polyunsaturated fatty acids. The administration of semaglutide to leptin-receptor-deficient, hyperphagic and diabetic mice resulted in the amelioration of MASLD, likely independently of daily caloric intake, suggesting a direct effect of semaglutide on the liver through modulation of the lipid profile.

## 1. Introduction

Metabolic-dysfunction-associated steatotic liver disease (MASLD) currently ranks as the most prevalent liver disorder globally, with a prevalence exceeding 30% and which is escalating at an alarming pace [1,2]. The main causes of MASLD predominantly stem from conditions such as obesity, metabolic syndrome and/or type 2 diabetes mellitus (T2DM) [3]. Diabetes and obesity, collectively termed as diabesity, induce fat accumulation in the liver, a hallmark frequently observed in MASLD patients [4,5]. Excessive ectopic lipid deposition promotes hepatic steatosis and can lead to metabolic-dysfunction-associated steatohepatitis (MASH), with or without fibrosis, and can potentially advance to cirrhosis, liver failure and hepatocarcinoma [6,7].

Given the close link between MASLD and T2DM, antidiabetic drugs such as glucagon-like peptide 1 (GLP-1) receptor modulators have been employed to prevent MASLD progression, achieving favorable outcomes in both preclinical models and patients cohorts [7,8,9]. GLP-1 primarily acts by stimulating postprandial insulin secretion from beta cells and inhibiting glucagon secretion from alpha cells in a glucose-dependent manner [10]. By improving tissue insulin sensitivity, GLP-1 receptor agonists (GLP-1RAs) help regulate systemic glucose levels, potentially mitigating MASLD or other diabetic complications [11,12]. GLP-1RAs also confer additional benefits such as slowing, including delayed gastric emptying and increasing gastrointestinal motility, leading to less glucose reabsorption, as well as promoting satiety via direct central nervous system action [13,14]. 

Although the presence of the GLP-1 receptor in the liver remains controversial, GLP-1 has demonstrated an indirect hepatoprotective effect via the gut–pancreas–liver axis [15,16]. Thus, the actions of GLP-1RAs involve multiple pathways, including decreased inflammation, heightened insulin sensitivity, inhibition of endoplasmic reticulum stress and improved mitochondrial function [17,18]. Consequently, the effect of GLP-1RAs on lipotoxicity and autophagy is associated to weight loss as well as other mechanisms that could be weight-independent, such as a direct effect on de novo lipogenesis [11,16,19].

Clinically, the STEPs study group recently showcased that semaglutide, a new generation of GLP-1 RAs, administered once-weekly via subcutaneous injection induced significant (>15%) weight in overweight/obese individuals with or without T2DM [20,21,22]. Furthermore, the findings from Newsome et al. demonstrated the efficacy of semaglutide in resolving early-stage MASLD (steatosis and inflammation), while its impact on advanced fibrosis and cirrhosis requires further research [23,24]. 

In this study, we employed a preclinical model of hyperphagia associated with diabesity development to elucidate the hepatoprotective mechanisms underlying semaglutide’s therapeutic efficacy. Our investigation particularly focused on analyzing metabolic biomarkers and hepatic markers of de novo lipogenesis, which play key roles in the transition from steatosis to MASH. 

## 2. Results

### 2.1. Semaglutide Improves Systemic Metabolic Parameters, Hepatic Function and Body Composition in BKS db/db Mice

**Figure 1:**
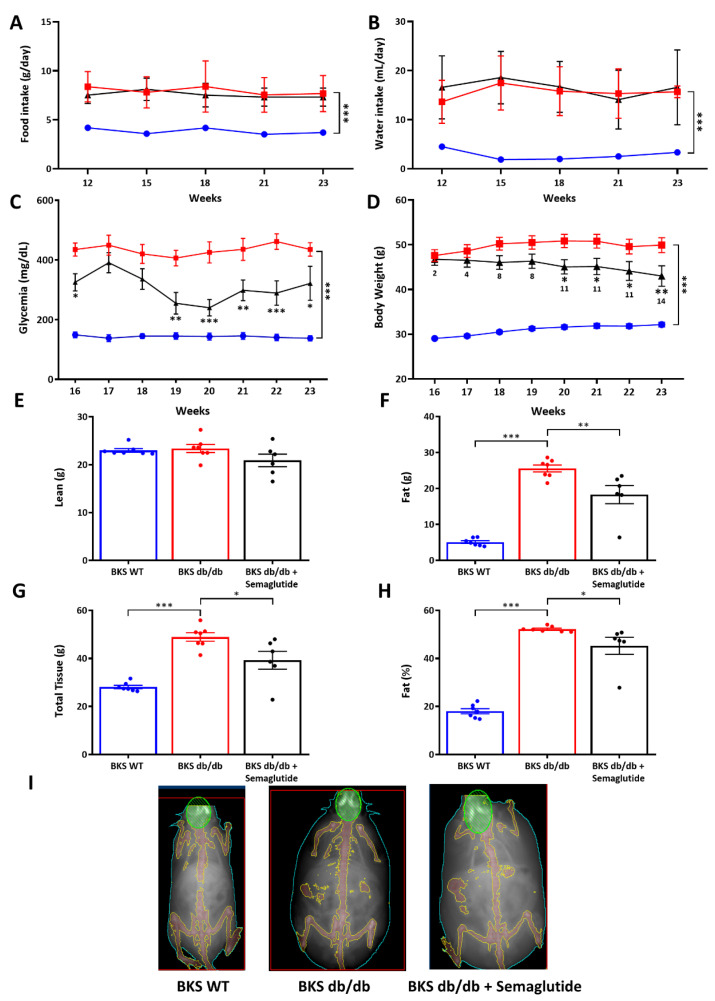
Food and water intake consumption, glycemia and body weight and body composition monitoring. Food (**A**) and water intake (**B**) between 12–23 weeks, non-fasting glycemia (**C**) and body weight (**D**) between 16–23 weeks in BKS WT (Blue), BKS db/db untreated (Red) and treated-with-semaglutide (Black)(25 μg/kg/week for 2 weeks followed by 100 μg/kg/week for 9 weeks) groups. The number under the semaglutide measurement indicates the percentage body weight reduction vs. BKS db/db. Analysis of lean mass (**E**), fat mass (**F**), total tissue (**G**), % fat (**H**) and representative images of DXA scanning (**I**) in the experimental model. Data are shown as scatter dot plots and mean ± SEM of each group (*n* = 6–8 mice/group); * *p* < 0.05, ** *p* < 0.01 and *** *p* < 0.0001 vs. BKS db/db.

In the BKS db/db mice, as expected, both the average daily food and water intake were augmented compared to the control group (BKS WT); however, there were no statistically significant differences between the untreated and semaglutide-treated BKS db/db groups (Figure 1A,B). Semaglutide significantly decreased non-fasting blood glucose levels from week 19 to week 23 (Figure 1C) and caused an 11–14% decrease in body weight (Figure 1D).

The semaglutide treatment also affected the body composition, as determined by DXA analysis. At 23 weeks of age, there was no alteration in lean mass (Figure 1E), but a significant reduction in fat mass (Figure 1F), total tissue mass (Figure 1G) and body fat percentage (Figure 1H) was observed in the semaglutide-treated BKS db/db mice compared to the untreated group. 

As outlined in Table 1, at the end of the study, the BKS db/db mice exhibited a substantial increase in non-fasting glycemia, body weight, liver weight and serum levels of liver enzymes (ALT, AST and AP) and lipids (TGs, total and HDL cholesterol) compared to the BKS WT mice, while the semaglutide treatment significantly lowered the levels of body weight, liver weight (−27%), AST (−33%), ALT (−62%), AP (−46%) and c-HDL (−16%).

### 2.2. Treatment with Semaglutide Ameliorates NAFLD Activity Score and Reduces Intrahepatic Lipids

**Figure 2:**
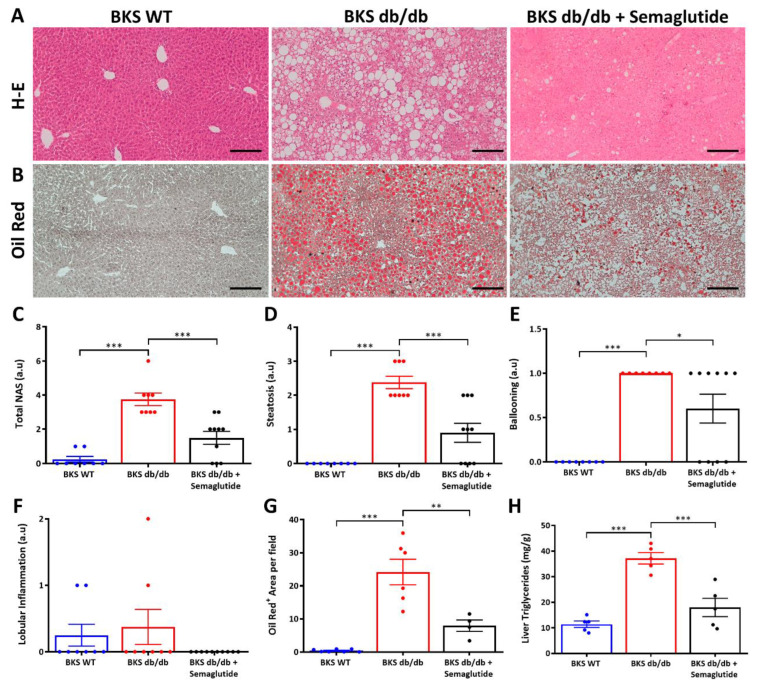
Liver histopathological changes in the experimental model. Representative images (100× magnification) of hematoxylin–eosin staining (**A**) and oil red O staining (**B**) in liver samples from BKS WT, untreated BKS db/db and semaglutide-treated BKS db/db mice (BKS db/db + semaglutide, treated with an induction dose of 25 μg/kg/week for 2 weeks followed by 100 μg/kg/week for 9 weeks). (**C**) Quantification of NAFLD activity score (total NAS) and its histopathological characteristics: steatosis (**D**), hepatocellular ballooning (**E**) and lobular inflammation (**F**). (**G**) Quantification of positive oil red O staining. (**H**) Quantification of liver TGs by colorimetric kit. Data are shown as scatter dot plots and mean ± SEM of each group (*n* = 6–8 mice/group); * *p* < 0.05, ** *p* < 0.01 and *** *p* < 0.001 vs. BKS db/db.

The liver morphological analysis was evaluated at the end of the study period (23rd week). The untreated BKS db/db mice showed characteristic features of MASLD, including micro- and macrovesicular steatosis, predominantly in zone 2 and 3 (transitional and pericentral, respectively), inflammatory clusters and hepatocellular ballooning, while fibrosis was not appreciated. These histological findings were ameliorated by the semaglutide treatment (Figure 2A). The results from the NAFLD activity score (NAS) assessment revealed that the semaglutide treatment markedly and significantly reduced total NAS (Figure 2C), steatosis (Figure 2D) and hepatocellular ballooning (Figure 2E) and also tended to decrease the number of inflammatory aggregates (Figure 2F). 

Additionally, to assess the intrahepatic lipid content, liver samples were stained with oil red O (Figure 2B). Quantification of positive staining per field (Figure 2G) revealed a significant increase in lipid droplets in the BKS db/db mice compared to the BKS WT mice, which were prominently attenuated by the semaglutide administration. These results were supported by measurement of the hepatic TG content using a colorimetric assay (Figure 2H).

### 2.3. Semaglutide Modifies Lipidomic Profile and De Novo Lipogenesis Markers in Liver of BKS db/db Mice

**Figure 3:**
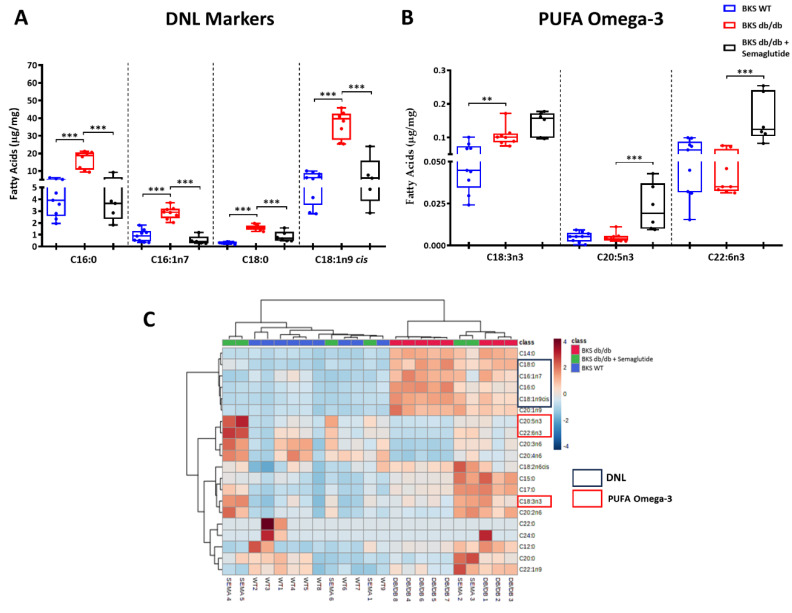
Lipid composition in liver at 23 weeks. Analysis of fatty acids in liver TG fractions by gas chromatography/electron ionization mass spectrometry (GC-MS). Concentrations of de novo lipogenesis markers (**A**) and omega-3 polyunsaturated fatty acids (PUFAs) (**B**). (**C**) Hierarchical clustering based on liver lipid composition showing the upregulated (dark brown) and downregulated (dark blue) lipids in each experimental group. The semaglutide group was treated with an induction dose of 25 μg/kg/week for 2 weeks followed by 100 μg/kg/week for 9 weeks. Data are shown as box plots and mean ± SEM of each group (*n* = 5–8 mice/group); ** *p* < 0.01 and *** *p* < 0.001 vs. BKS db/db. Abbreviations: DNL: de novo lipogenesis.

To further investigate the link between MASLD and de novo lipogenesis, we examined the specific fatty acids comprising the intrahepatic TG fraction. Compared to the BKS WT mice, the BKS db/db mice showed a significant increase in the end products of de novo lipogenesis such as palmitic acid (C16:0; 407%), palmitoleic acid (C16:1n7; 303%), stearic acid (C18:0; 492%) and oleic acid (C18:1n9 cis; 587%). It is noteworthy that these differences were reduced by 32–50% following the treatment with semaglutide (Figure 3A). In the semaglutide-treated BKS db/db group, there was also an increase in omega-3 polyunsaturated fatty acids (omega-3 PUFAs), including alpha-linolenic acid (C18:3n3), eicosapentaenoic acid (C20:5n3) and docosahexaenoic acid (C22:6n3) (Figure 3B). Hierarchical clustering revealed distinctive lipid profiles associated with lipid composition in each of the three groups (Figure 3C). Detailed information regarding the composition of the TG fraction in saturated fatty acids, mono-unsaturated fatty acids and PUFAs is provided in Supplementary Table S1.

**Figure 4:**
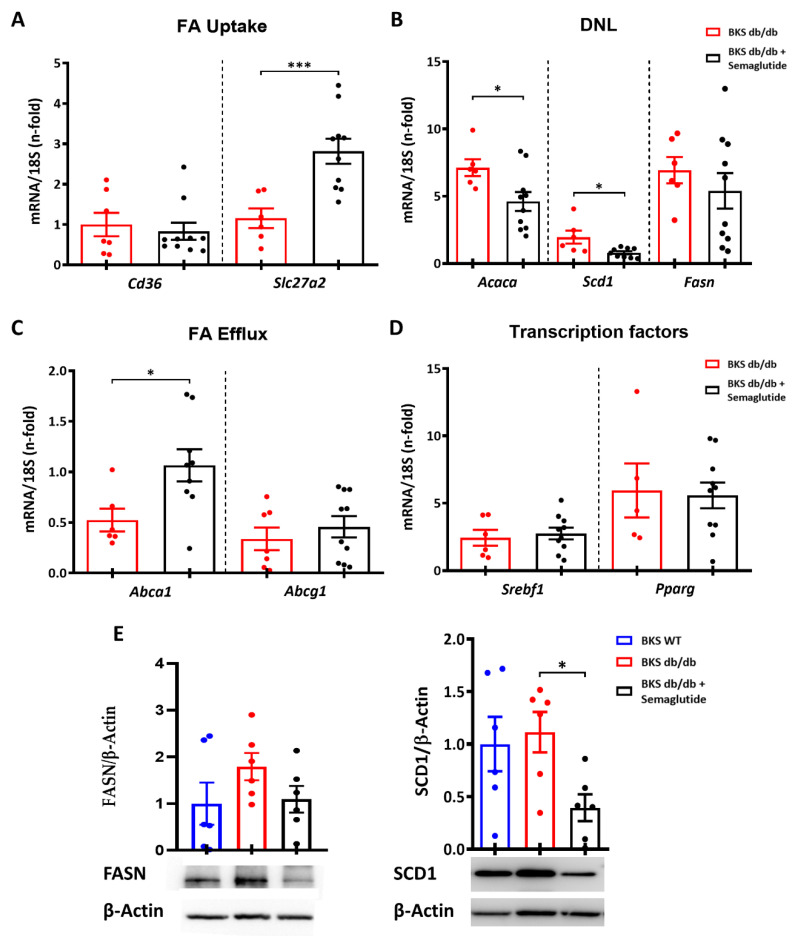
Gene and protein expression of markers related to hepatic lipid metabolism. RNA expression analysis of genes involved in fatty acid uptake (CD36, Slc27a2) (**A**), de novo lipogenesis (Acaca, Fasn, Scd1) (**B**), fatty acid efflux (Abca1, Abcg1) (**C**) and transcription factors (Srebf1, Pparg) (**D**). The qPCR values were normalized by 18S rRNA and expressed as fold increases compared to BKS WT group. (**E**) Protein expression of lipogenic enzymes was evaluated by Western blot. Fold-change levels of proteins normalized by β-Actin and images of their respective Western blot. The semaglutide group was treated with an induction dose of 25 μg/kg/week for 2 weeks followed by 100 μg/kg/week for 9 weeks. Data are shown as box plots and mean ± SEM of each group (*n* = 6–10 mice/group); * *p* < 0.05 and *** *p* < 0.01 vs. BKS db/db. Abbreviations: FA: fatty acids; DNL: de novo lipogenesis.

We then sought to investigate the impact of the semaglutide treatment on the liver by focusing on the following different signaling pathways involved in hepatic lipid metabolism: fatty acids uptake (FA uptake), de novo lipogenesis, fatty acids efflux (FA efflux) and transcription factors. Real-time PCR analysis revealed an increased expression of *Slc27a2* fatty acid uptake transporter, but no changes in the *Cd36* receptor were observed in the BKS db/db mice treated with semaglutide. (Figure 4A). Regarding de novo lipogenesis, the semaglutide-treated BKS db/db mice showed significant downregulation of the *Acaca* and *Scd1* genes (Figure 4B). The semaglutide treatment also resulted in increased FA efflux transport (*Abca1*) (Figure 4C) but had no effect on transcription factors altered in lipid metabolism in the BKS db/db mice (Figure 4D). Furthermore, the reduction in de novo lipogenesis markers observed after the semaglutide treatment was confirmed at the protein level by Western blot analysis, showing a significant decrease in SCD1 expression. (Figure 4E). 

## 3. Discussion

Our study reveals that the administration of semaglutide to leptin receptor-deficient hyperphagic diabetic mice results in the amelioration of MASLD, likely independent of food intake, suggesting a direct effect of semaglutide on the liver. The semaglutide treatment reduced glycemia, body weight, serum markers of liver dysfunction and both total and percentage of fat mass in the BKS db/db mice. Histologically, the semaglutide decreased hepatic steatosis, hepatocellular ballooning, intrahepatic triglycerides and de novo lipogenesis markers and modified lipid composition, resulting in an increase in polyunsaturated fatty acids.

Currently, the etiopathological factors contributing to the development and progression of MASLD are complex, heterogeneous and not fully elucidated so far. Although none of the potential anti-MASLD drugs have been approved by international drug agencies to date, recent clinical trials have demonstrated that GLP1-RAs, particularly semaglutide, exert specific beneficial effects on this clinical condition. 

In the hyperphagic leptin-receptor-deficient BKS db/db mice, an experimental model resembling human diabesity, we observed that the administration of semaglutide for 11 weeks led to a decrease in average body weight, which was consistent with the reported effect of this drug in obesity and diabetes contexts [25]. Consequently, there was a significant reduction in blood glucose and body composition (total fat mass and fat mass percentage) along with a normalization in liver dysfunction markers, mainly ALT and AP. These changes were associated with a marked attenuation of histological MASLD in the BKS db/db mice, akin to observations in clinical studies [26].

The main effect of the incretin mimetics as antidiabetic drugs resides in their capacity to regulate glucose levels by stimulating insulin release and suppressing glucagon secretion from pancreatic cells in response to food intake [14,27]. Recent clinical studies have highlighted the importance of GLP-1RAs as a therapeutic approach to combat the rising prevalence of obesity worldwide and to mitigate the incidence of MASLD [20,21].

Indeed, reductions in body weight have been observed in both animal and human studies, primarily attributed to decreased caloric intake and delayed gastric emptying, leading to increased satiety [14,28]. However, our findings indicate a reduction in body weight without a significant decrease in food consumption. This outcome is consistent, to some extent, with the results reported by Inia et al., who observed a body weight reduction in Ldlr-/- obese mice following 12 weeks of semaglutide treatment, despite a significant decrease in average daily intake only during the initial two weeks of treatment, with no sustained reduction afterwards [29]. A similar decrease in body weight was reported in a diet-induced obese murine model [30], where a reduction in food intake was observed only during the first 10 out 77 days of semaglutide administration.

While previous studies using Exendin-4 or semaglutide displayed weight loss and reduced hepatic steatosis in various models of fatty liver [31,32,33], the underlaying mechanisms remain not fully unveiled. The experimental model used in our study, hyperphagic and diabetic BKS db/db mice, is recognized as a reliable model for studying the complications of type 2 diabetes, and similar studies have been performed to investigate MASLD [34]. Given the close association between MASLD and diabetes progression, this model constitutes an excellent approach to study the potential mechanisms involved on the effects of semaglutide on fatty liver. In our model, the semaglutide treatment markedly and significantly reduced steatosis lesions, hepatocellular ballooning and inflammatory aggregates, which are hallmarks of MASLD progression. 

Elevated hepatic de novo lipogenesis, a process in which the liver synthesizes fatty acids from dietary carbohydrates and proteins, is among the earliest metabolic abnormalities related to insulin resistance and fatty liver disease [35]. In this context, we observed that semaglutide led to a reduction in intrahepatic TG content and de novo lipogenesis, as evidenced by decreased levels of de novo lipogenesis end products, such as lipotoxic fatty acids (palmitic, palmitoleic and oleic acids) and the downregulation of lipogenic enzymes (Acaca, Fasn and Scd1). These changes were accompanied/followed by an improvement in circulating TGs. Among the de novo lipogenesis mediators downregulated by the semaglutide treatment, we found Scd1, a desaturase predominantly expressed in hepatocytes and adipocytes, which allows for the conversion of saturated fatty acids to monounsaturated fatty acids. Inhibition of Scd1 has been associated with improvements in hepatic steatosis [36,37]. Indeed, total or liver-specific SCD1 knockout mice are partially protected from diet-induced obesity and MASLD development [38,39]. Semaglutide notably reduced the gene and expression levels of Scd1 in the liver of the BKS db/db mice, suggesting a direct effect of semaglutide on hepatic lipogenesis, consistent with findings observed with liraglutide [40]. Some data suggest that this effect could be exerted via the AMPK pathway, as proposed in other studies involving GLP-1 RA [41] or with semaglutide itself in another obesity model [33]. Taken together, these findings are considered significant for preventing MASLD progression, as modulation of the proteins involved in de novo lipogenesis targeted by semaglutide is considered a therapeutic approach [42,43]. 

Semaglutide also appears to modulate lipid metabolism by modifying fatty acid transport. Previous studies have suggested that in the early stages of SLD, there is an increase in fatty acid secretion to counteract hepatic lipid accumulation, but this output subsequently stabilizes or even decreases [44]. Our study demonstrates that semaglutide upregulated ABCA1 expression, a transporter involved in cholesterol efflux, which may play a role in preventing lipid accumulation in obese mice, as previously described [45]. Additionally, with regard to fatty acid uptake, we observed an augmentation in the expression of Slc27a2 (FATP2) in the BKS db/db mice treated with semaglutide. While FATP2 is recognized as a fatty acid transporter, it also possesses the capability to generate CoA derivatives of n-3 fatty acids, which are preferentially trafficked in phosphatidylinositol, potentially explaining our observation [46]. 

Although the reduction in insulin resistance and the reversal of hepatic fat accumulation has been attributed to the effects of GLP-1RAs on satiety, our study showed notable effects, both quantitatively and qualitatively, on hepatic lipid composition, likely independently of changes in food intake. This suggests an additional direct action of semaglutide on liver cells, as previously reported in other animal models [47]. 

Interestingly, the quantitative lipidomic analysis also revealed that semaglutide increased the production of omega-3 PUFAs (α-linoleic, eicosapentaenoic and docosahexaenoic acids) in the liver, fatty acids known for their potent anti-inflammatory and hepatoprotective effect [31,40]. It has been demonstrated that there exists a relationship between PUFAs and GLP-1, in which treatment with omega-3 derivatives is capable of stimulating GLP-1 secretion [48], though there is limited evidence that the administration of a GLP-1 RA could be have some effect on the PUFA content [49]. This local action on the liver could exert systemic effects or additive non-insulinotropic actions in diseases such as obesity, type 2 diabetes mellitus and its macro/microvascular complications, which could be considered important in preventing the progression of MASLD [42,43].

## 4. Material and Methods

### 4.1. Experimental Model

The effects of semaglutide on the liver were assessed in leptin-receptor-deficient db/db mice crossed with the BKS strain (BKS.Cg-+Leprdb/+Leprdb/OlaHsd), which developed complications of diabesity, including MASLD [34]. Male mice were divided into 3 groups (Supplementary Figure S1): (1) control non-diabetic (BKS.Cg-(Lean)/OlaHsd) wild-type mice (BKS WT, *n* = 8); (2) untreated obese and diabetic mice (BKS db/db, *n* = 8); and (3) obese and diabetic mice treated with semaglutide (Novo Nordisk, Bagsværd, Denmark) diluted in saline solution and injected subcutaneously once weekly at an induction dose of 25 μg/kg/week for two weeks followed by a therapeutic dose of 100 μg/kg/week for an additional 9 weeks (BKS db/db + semaglutide, *n* = 10). The therapeutic dose was defined based on a dose analysis evaluated in clinical trials (STEP-1 study; 24 µg/kg/week; equivalent to 2.4 mg in patients weighing 100 kg) [20] and preclinical studies (4, 12, 60 µg/kg/week in ApoE^−/−^ and LDLr^−/−^ mice; 1–100 nmol/kg/day in diet-induced obese mice and rats) [30,50].

Treatment started at 12 weeks of age and continued until 23 weeks, after which the mice were euthanized under anesthesia (ketamine 100 mg/kg and xylazine 10 mg/kg) and blood and liver samples were collected. The treated mice were housed at a density of two animals per cage in individually ventilated cages (20–22 °C) with 12 h light–dark cycles and were fed ad libitum with a standard diet (3.5% fat content). Both food and water intake were measured every other 3rd week from 12 weeks of age until sacrifice. Glycemia and body weight were measured weekly, one day after injection, using a NovaPro glucometer (Nova Biomedical Iberia, Barcelona, Spain) and digital balance, respectively. The animal studies were approved by the FISS-FJD Animal Experimentation Ethics Committee and by the Madrid regional government (Ref. PROEX 079/18). All animal procedures conformed to EU Directive 2010/63 EU and national regulations (RD 53/2013) regarding the protection of animals used for experimental and scientific purposes. 

### 4.2. Biochemical Parameters

Serum lipid profile and liver biochemical parameters, including triglycerides (TGs), total cholesterol, high-density lipoprotein (HDL) cholesterol, aspartate transaminase (AST), alanine transaminase (ALT) and alkaline phosphatase (AP), were assessed using a Roche Cobas autoanalyzer at the central laboratories of our institution. 

### 4.3. Dual-Energy X-ray Absorptiometry (DXA)

Body composition was assessed at 23 weeks using a PIXImus2 bone densitometer installed with software version 1.46 (GE Medical Systems, Bedford, UK). Data on lean mass, fat mass, total tissue and % fat were quantified by using the PIXImus software. The instrument was calibrated before each scanning session using a phantom with a known body mineral density according to the manufacturer’s guidelines. The mice were anesthetized with isoflurane and placed in the prone position on the sample tray to scan the whole body (*n* = 6–7). 

### 4.4. Liver Morphological Analysis

A piece of liver was fixed in 4% formaldehyde and further embedded in paraffin. Tissue sections (4 µm) were stained with hematoxylin–eosin for histochemical studies. Liver damage was semiquantitative assessed by trained personnel in a blinded manner according to the NAFLD activity score (NAS) described by Kleiner et al. [51]. Oil red O staining was performed in OCT-frozen samples (8 µm sections, formalin-fixed) followed by hematoxylin counterstaining. Positive staining was quantified using the Image-Pro Plus software v7 (Media Cybernetics, Rockville, MD, USA) and expressed as a percentage of the positive area per field.

### 4.5. Lipid Profile Determination

Liver homogenates were obtained from a weighed amount of tissue (20 mg). Fatty acid methyl esters from liver TGs were determined by gas chromatography/electron ionization mass spectrometry after solid-phase extraction, as previously described [52].

### 4.6. Gene Expression Studies

Liver tissue was disaggregated using 1 mm diameter zirconium beads and a homogenizer (Bullet Blender Homogenizer, Next Advance Inc., Troy, NY, USA). Total RNA was isolated with TRIdity G A4051 (Panreac AppliChem, Barcelona, Spain) and quantified by a NanoPhotometer^®^ N60 (Implen Inc., Westlake Village, CA, USA). Complementary DNA (cDNA) was synthesized using a High-Capacity cDNA Archive Kit (Applied Biosystems, Foster City, CA, USA) with 2 µg of total RNA primed with random primers following the manufacturer’s instructions. Quantitative gene expression analysis was performed by RT-qPCR (quantitative real-time PCR 7500 Applied Biosystems, System SDS software V.1.2b1c3) using TaqMan gene expression assays. The mouse assay IDs were as follows: Cd36 (Mm01135198_m1), *Slc27a2* (fatty acid transporter 2 (FATP2); Mm00449517_m1), *Acaca* (acetyl-Coenzyme A carboxylase alpha; Mm01304289_m1), *Fasn* (fatty acid synthase; Mm00662319_m1), *Scd1* (stearoyl-Coenzyme A desaturase 1; Mm00772290_m1), *Abca1* (ATP-binding cassette, sub-family A member 1; Mm00442646_m1), *Abcg1* (ATP-binding cassette, sub-family G member 1; Mm00437390_m1), *Srebf1* (sterol regulatory element binding transcription factor 1 (SREBP1); Mm00550338_m1) and *Pparg* (peroxisome-proliferator-activated receptor gamma (PPAR-γ); Mm00440940_m1). Target gene expression was analyzed in duplicate and normalized to the housekeeping gene *18s* rRNA (4310893E). Gene expression results are represented as fold changes relative to the BKS WT group. 

### 4.7. Protein Studies

All chemical reagents have been purchased from Sigma-Aldrich (Saint Louis, MO, USA) unless otherwise stated. Liver tissue samples were homogenized in a Bullet Blender Homogenizer (Next Advance, Inc., Troy, NY, USA) with lysis buffer (50 mM Tris–HCl, 150 mM of NaCl, 2 mM of EDTA, 2 mM of EGTA, 0.2% Triton X-100, 0.3% Igepal) complemented with protease and phosphatase inhibitor cocktails (CP8340 and P0044). Proteins were quantified using a BCA protein assay kit (Thermo Fisher Scientific, Waltham, MA, USA), and equal amounts (50 μg) were resolved on 8–12% polyacrylamide gels under reducing conditions. Following electrophoresis, the samples were transferred to PVDF membranes (IPVH00010, Millipore, Bedford, MA, USA), blocked in TBS containing 0.1% Tween 20 (TBS-T) and 5% skimmed milk for 1 h at room temperature and incubated overnight at 4 °C with the following primary antibodies (1:1000 dilution): FASN (C20G5) (Cat#3180S, RRID:AB_2100796, Cell Signaling Technology; Danvers, MA, USA) and SCD1 (C12H5) (Cat#2794S, RRID:AB_2183099, Cell Signaling Technology). Membranes were subsequently incubated with peroxidase-conjugated IgG secondary antibody (anti-mouse or anti-rabbit; 1:2500 dilution; Invitrogen, Waltham, MA, USA) and developed using ECL chemiluminescence (ECL Luminata Crescendo, WBLUR05000). Loading controls were performed using a mouse monoclonal anti-β-Actin antibody (1:5000 dilution, A2228, Sigma-Aldrich). The results were analyzed by an iBright™ CL750 Imaging System (Invitrogen) and quantified using the Quantity One software https://www.bio-rad.com/es-es/product/quantity-one-1-d-analysis-software?ID=1de9eb3a-1eb5-4edb-82d2-68b91bf360fb (Bio-Rad, Hercules, CA, USA).

### 4.8. Statistical Analysis

The data are presented as bar plots with mean ± SEM (graphs) or median ± IQR (tables) of the total number of animals. The graphs and corresponding statistical tests were performed by the GraphPad Prism V.8 software. Statistical analyses were performed using one-way or two-way ANOVA followed by the corresponding post hoc analyses or the unpaired Mann–Whitney U-test, as appropriate.

## 5. Conclusions

Semaglutide improves metabolic control, body composition and body weight in BKS db/db mice by reducing hepatic steatosis, lipotoxic fatty acid synthesis and markers of liver damage. This beneficial effect could be, at least in part, due to the modulation of intrahepatic de novo lipogenesis, a key mechanism in the transition from steatosis to steatohepatitis. 

This research strengthens the metabolic effects of semaglutide and supports its clinical benefits in patients with fatty liver disease.

## Figures and Tables

**Table 1:** Metabolic and biochemical parameters in the experimental model. Data are shown as median (IQR) (*n* = 6–10 mice/group). # *p* < 0.05; ### *p* < 0.001 vs. BKS WT and * *p* < 0.05; ** *p* < 0.01; *** *p* < 0.001 vs. BKS db/db. Semaglutide group was treated with an induction dose of 25 μg/kg/week for 2 weeks followed by 100 μg/kg/week for 9 weeks.

Variables	BKS WT	BKS db/db	BKS db/db + Semaglutide
Glycemia (mg/dL)	**164.0** (155.8–176.8)	**431.0** (374.8–502.3) ^###^	**308.0** (158.3–490.0) *
Body Weight (g)	**31.7** (30.9–33.5)	**50.3** (46.0–53.9) ^###^	**42.2** (38.6–48.6) **
Liver Weight (g)	**1.4** (1.1–1.8)	**5.2** (4.6–5.3) ^###^	**3.8** (2.9–4.1) ***
AST (IU/L)	**95.0** (65.0–129.0)	**140.0** (117.0–241.0)	**94.0** (82.5–119.8) *
ALT (IU/L)	**42.0** (32.0–48.0)	**195.0** (179.0–318.0) ^###^	**74.0** (60.3–113.8) ***
AP (IU/L)	**83.5** (70.8–94.8)	**211.0** (180.0–257.0) ^###^	**113.0** (106.0–134.0) ***
TGs (mg/dL)	**73.5** (54.3–84.3)	**94.0** (78.0–198.0) ^#^	**65.0** (47.0–143.0)
Total Cholesterol (mg/dL)	**88.0** (82.2–91.2)	**165.0** (145.3–192.2) ^###^	**140.0** (129.0–171.0)
c-HDL (mg/dL)	**85.0** (78.0–92.0)	**135.0** (121.0–154.3) ^###^	**113.0** (106.5–138.5) *

## Data Availability

Data are contained within the article and Supplementary Materials.

## Supplementary Materials

The following supporting information can be downloaded at: https://www.mdpi.com/article/10.3390/ijms25052961/s1.

## Abbreviations

Abca1	ATP-binding cassette, sub-family A member 1
ALT	alanine transaminase
AST	aspartate transaminase
AP	alkaline phosphatase
c-HDL	high-density lipoprotein cholesterol
Diabesity	diabetes and obesity
DXA	dual-energy X-ray absorptiometry
FA	fatty acid
FATP	fatty acid transport protein
GLP1-RA	glucagon-like peptide 1 receptor agonist
MASLD	metabolic-dysfunction-associated steatotic liver disease
MASH	metabolic-dysfunction-associated steatohepatitis
NAFLD	non-alcoholic fatty liver disease
NAS	NAFLD activity score
PUFA	polyunsaturated fatty acid
SCD1	Stearoyl-CoA desaturase-1
T2DM	type 2 diabetes mellitus
TGs	triglycerides

## References

[1] Riazi K., Azhari H., Charette J.H., Underwood F.E., King J.A., Afshar E.E., Swain M.G., Congly S.E., Kaplan G.G., Shaheen A.A. (2022). The prevalence and incidence of NAFLD worldwide: A systematic review and meta-analysis. Lancet Gastroenterol. Hepatol..

[2] Younossi Z.M. (2019). Non-alcoholic fatty liver disease—A global public health perspective. J. Hepatol..

[3] Rinella M.E., Lazarus J.V., Ratziu V., Francque S.M., Sanyal A.J., Kanwal F., Romero D., Abdelmalek M.F., Anstee Q.M., Arab J.P. (2023). A multi-society Delphi consensus statement on new fatty liver disease nomenclature. Hepatology.

[4] Targher G., Corey K.E., Byrne C.D., Roden M. (2021). The complex link between NAFLD and type 2 diabetes mellitus—Mechanisms and treatments. Nat. Rev. Gastroenterol. Hepatol..

[5] Sung K.C., Jeong W.S., Wild S.H., Byrne C.D. (2012). Combined Influence of Insulin Resistance, Overweight/Obesity, and Fatty Liver as Risk Factors for Type 2 Diabetes. Diabetes Care.

[6] McPherson S., Hardy T., Henderson E., Burt A.D., Day C.P., Anstee Q.M. (2015). Evidence of NAFLD progression from steatosis to fibrosing-steatohepatitis using paired biopsies: Implications for prognosis and clinical management. J. Hepatol..

[7] Ferguson D., Finck B.N. (2021). Emerging therapeutic approaches for the treatment of NAFLD and type 2 diabetes mellitus. Nat. Rev. Endocrinol..

[8] Somm E., Montandon S.A., Loizides-Mangold U., Gaïa N., Lazarevic V., De Vito C., Perroud E., Bochaton-Piallat M.L., Dibner C., Schrenzel J. (2021). The GLP-1R agonist liraglutide limits hepatic lipotoxicity and inflammatory response in mice fed a methionine-choline deficient diet. Transl. Res. J. Lab. Clin. Med..

[9] Dutour A., Abdesselam I., Ancel P., Kober F., Mrad G., Darmon P., Ronsin O., Pradel V., Lesavre N., Martin J.C. (2016). Exenatide decreases liver fat content and epicardial adipose tissue in patients with obesity and type 2 diabetes: A prospective randomized clinical trial using magnetic resonance imaging and spectroscopy. Diabetes. Obes. Metab..

[10] Rajeev S.P., Wilding J. (2016). GLP-1 as a target for therapeutic intervention. Curr. Opin. Pharmacol..

[11] Lee H.A., Kim H.Y. (2023). Therapeutic Mechanisms and Clinical Effects of Glucagon-like Peptide 1 Receptor Agonists in Nonalcoholic Fatty Liver Disease. Int. J. Mol. Sci..

[12] Mells J.E., Fu P.P., Sharma S., Olson D., Cheng L., Handy J.A., Saxena N.K., Sorescu D., Anania F.A. (2012). Glp-1 analog, liraglutide, ameliorates hepatic steatosis and cardiac hypertrophy in C57BL/6J mice fed a Western diet. Am. J. Physiol. Gastrointest. Liver Physiol..

[13] Aldawsari M., Almadani F.A., Almuhammadi N., Algabsani S., Alamro Y., Aldhwayan M. (2023). The Efficacy of GLP-1 Analogues on Appetite Parameters, Gastric Emptying, Food Preference and Taste Among Adults with Obesity: Systematic Review of Randomized Controlled Trials. Diabetes Metab. Syndr. Obesity Targets Ther..

[14] Cornell S. (2020). A review of GLP-1 receptor agonists in type 2 diabetes: A focus on the mechanism of action of once-weekly agents. J. Clin. Pharm. Ther..

[15] Panjwani N., Mulvihill E.E., Longuet C., Yusta B., Campbell J.E., Brown T.J., Streutker C., Holland D., Cao X., Baggio L.L. (2013). GLP-1 receptor activation indirectly reduces hepatic lipid accumulation but does not attenuate development of atherosclerosis in diabetic male *ApoE*^−/−^ mice. Endocrinology.

[16] Mahapatra M.K., Karuppasamy M., Sahoo B.M. (2022). Therapeutic Potential of Semaglutide, a Newer GLP-1 Receptor Agonist, in Abating Obesity, Non-Alcoholic Steatohepatitis and Neurodegenerative diseases: A Narrative Review. Pharm. Res..

[17] Patel Chavez C., Cusi K., Kadiyala S. (2022). The Emerging Role of Glucagon-like Peptide-1 Receptor Agonists for the Management of NAFLD. J. Clin. Endocrinol. Metab..

[18] Gastaldelli A., Cusi K. (2019). From NASH to diabetes and from diabetes to NASH: Mechanisms and treatment options. JHEP Rep. Innov. Hepatol..

[19] Sharma S., Mells J.E., Fu P.P., Saxena N.K., Anania F.A. (2011). GLP-1 analogs reduce hepatocyte steatosis and improve survival by enhancing the unfolded protein response and promoting macroautophagy. PLoS ONE.

[20] Wilding J.P.H., Batterham R.L., Calanna S., Davies M., Van Gaal L.F., Lingvay I., McGowan B.M., Rosenstock J., Tran M.T.D., Wadden T.A. (2021). Once-Weekly Semaglutide in Adults with Overweight or Obesity. N. Engl. J. Med..

[21] Garvey W.T., Batterham R.L., Bhatta M., Buscemi S., Christensen L.N., Frias J.P., Jódar E., Kandler K., Rigas G., Wadden T.A. (2022). Two-year effects of semaglutide in adults with overweight or obesity: The STEP 5 trial. Nat. Med..

[22] Rubino D.M., Greenway F.L., Khalid U., O’Neil P.M., Rosenstock J., Sørrig R., Wadden T.A., Wizert A., Garvey W.T., for the STEP 8 Investigators (2022). Effect of Weekly Subcutaneous Semaglutide vs Daily Liraglutide on Body Weight in Adults with Overweight or Obesity without Diabetes: The STEP 8 Randomized Clinical Trial. JAMA.

[23] Newsome P.N., Buchholtz K., Cusi K., Linder M., Okanoue T., Ratziu V., Sanyal A.J., Sejling A.-S., Harrison S.A. (2021). A Placebo-Controlled Trial of Subcutaneous Semaglutide in Nonalcoholic Steatohepatitis. N. Engl. J. Med..

[24] Loomba R., Abdelmalek M.F., Armstrong M.J., Jara M., Kjær M.S., Krarup N., Lawitz E., Ratziu V., Sanyal A.J., Schattenberg J.M. (2023). Semaglutide 2·4 mg once weekly in patients with non-alcoholic steatohepatitis-related cirrhosis: A randomised, placebo-controlled phase 2 trial. Lancet Gastroenterol. Hepatol..

[25] Davies M., Færch L., Jeppesen O.K., Pakseresht A., Pedersen S.D., Perreault L., Rosenstock J., Shimomura I., Viljoen A., Wadden T.A. (2021). Semaglutide 2·4 mg once a week in adults with overweight or obesity, and type 2 diabetes (STEP 2): A randomised, double-blind, double-dummy, placebo-controlled, phase 3 trial. Lancet.

[26] Loomba R., Sanyal A.J., Kowdley K.V., Terrault N., Chalasani N.P., Abdelmalek M.F., McCullough A.J., Shringarpure R., Ferguson B., Lee L. (2019). Factors Associated with Histologic Response in Adult Patients with Nonalcoholic Steatohepatitis. Gastroenterology.

[27] Singh S., Wright E.E., Kwan A.Y.M., Thompson J.C., Syed I.A., Korol E.E., Waser N.A., Yu M.B., Juneja R. (2017). Glucagon-like peptide-1 receptor agonists compared with basal insulins for the treatment of type 2 diabetes mellitus: A systematic review and meta-analysis. Diabetes Obes. Metab..

[28] Gutzwiller J.P., Drewe J., Göke B., Schmidt H., Rohrer B., Lareida J., Beglinger C. (1999). Glucagon-like peptide-1 promotes satiety and reduces food intake in patients with diabetes mellitus type 2. Am. J. Physiol..

[29] Inia J.A., Stokman G., Morrison M.C., Worms N., Verschuren L., Caspers M.P.M., Menke A.L., Petitjean L., Chen L., Petitjean M. (2023). Semaglutide Has Beneficial Effects on Non-Alcoholic Steatohepatitis in Ldlr^−/−^.Leiden Mice. Int. J. Mol. Sci..

[30] Gabery S., Salinas C.G., Paulsen S.J., Ahnfelt-Rønne J., Alanentalo T., Baquero A.F., Buckley S.T., Farkas E., Fekete C., Frederiksen K.S. (2020). Semaglutide lowers body weight in rodents via distributed neural pathways. JCI Insight.

[31] Ding X., Saxena N.K., Lin S., Gupta N., Anania F.A. (2006). Exendin-4, a Glucagon-Like Protein-1 (GLP-1) Receptor Agonist, Reverses Hepatic Steatosis in ob/ob Mice. Hepatology.

[32] Niu S., Chen S., Chen X., Ren Q., Yue L., Pan X., Zhao H., Li Z., Chen X. (2022). Semaglutide ameliorates metabolism and hepatic outcomes in an NAFLD mouse model. Front. Endocrinol..

[33] Pontes-da-Silva R.M., de Souza Marinho T., de Macedo Cardoso L.E., Mandarim-de-Lacerda C.A., Aguila M.B. (2021). Obese mice weight loss role on nonalcoholic fatty liver disease and endoplasmic reticulum stress treated by a GLP-1 receptor agonist. Int. J. Obes..

[34] Michurina S.V., Ishenko I.J., Klimontov V.V., Archipov S.A., Myakina N.E., Cherepanova M.A., Zavjalov E.L., Koncevaya G.V., Konenkov V.I. (2016). Linagliptin alleviates fatty liver disease in diabetic db/db mice. World J. Diabetes.

[35] Cross E., Dearlove D.J., Hodson L. (2023). Nutritional regulation of hepatic de novo lipogenesis in humans. Curr. Opin. Clin. Nutr. Metab. Care.

[36] Jeyakumar S.M., Vajreswari A. (2022). Stearoyl-CoA desaturase 1: A potential target for non-alcoholic fatty liver disease?-perspective on emerging experimental evidence. World J. Hepatol..

[37] Wang W., Kong Y., Wang X., Wang Z., Tang C., Li J., Yang Q., Chen Y.Q., Zhu S. (2023). Identification of novel SCD1 inhibitor alleviates nonalcoholic fatty liver disease: Critical role of liver-adipose axis. Cell Commun. Signaling CCS.

[38] Ntambi J.M., Miyazaki M., Stoehr J.P., Lan H., Kendziorski C.M., Yandell B.S., Song Y., Cohen P., Friedman J.M., Attie A.D. (2002). Loss of stearoyl–CoA desaturase-1 function protects mice against adiposity. Proc. Natl. Acad. Sci. USA.

[39] Miyazaki M., Flowers M.T., Sampath H., Chu K., Otzelberger C., Liu X., Ntambi J.M. (2007). Hepatic Stearoyl-CoA Desaturase-1 Deficiency Protects Mice from Carbohydrate-Induced Adiposity and Hepatic Steatosis. Cell Metab..

[40] Armstrong M.J., Hull D., Guo K., Barton D., Hazlehurst J.M., Gathercole L.L., Nasiri M., Yu J., Gough S.C., Newsome P.N. (2016). Glucagon-like peptide 1 decreases lipotoxicity in non-alcoholic steatohepatitis. J. Hepatol..

[41] Ben-Shlomo S., Zvibel I., Shnell M., Shlomai A., Chepurko E., Halpern Z., Barzilai N., Oren R., Fishman S. (2011). Glucagon-like peptide-1 reduces hepatic lipogenesis via activation of AMP-activated protein kinase. J. Hepatol..

[42] Xu X., Poulsen K.L., Wu L., Liu S., Miyata T., Song Q., Wei Q., Zhao C., Lin C., Yang J. (2022). Targeted therapeutics and novel signaling pathways in non-alcohol-associated fatty liver/steatohepatitis (NAFL/NASH). Signal Transduct. Target. Ther..

[43] Santos-Laso A., Gutiérrez-Larrañaga M., Alonso-Peña M., Medina J.M., Iruzubieta P., Arias-Loste M.T., López-Hoyos M., Crespo J. (2022). Pathophysiological Mechanisms in Non-Alcoholic Fatty Liver Disease: From Drivers to Targets. Biomedicines.

[44] Ipsen D.H., Lykkesfeldt J., Tveden-Nyborg P. (2018). Molecular mechanisms of hepatic lipid accumulation in non-alcoholic fatty liver disease. Cell. Mol. Life Sci..

[45] Cavelier C., Lorenzi I., Rohrer L., von Eckardstein A. (2006). Lipid efflux by the ATP-binding cassette transporters ABCA1 and ABCG1. Biochim. Biophys. Acta.

[46] Melton E.M., Cerny R.L., Watkins P.A., DiRusso C.C., Black P.N. (2011). Human Fatty Acid Transport Protein 2a/Very Long Chain Acyl-CoA Synthetase 1 (FATP2a/Acsvl1) Has a Preference in Mediating the Channeling of Exogenous n-3 Fatty Acids into Phosphatidylinositol. J. Biol. Chem..

[47] Svegliati-Baroni G., Saccomanno S., Rychlicki C., Agostinelli L., de Minicis S., Candelaresi C., Faraci G., Pacetti D., Vivarelli M., Nicolini D. (2011). Glucagon-like peptide-1 receptor activation stimulates hepatic lipid oxidation and restores hepatic signalling alteration induced by a high-fat diet in nonalcoholic steatohepatitis. Liver Int..

[48] Bhaswant M., Poudyal H., Brown L. (2015). Mechanisms of enhanced insulin secretion and sensitivity with n-3 unsaturated fatty acids. J. Nutr. Biochem..

[49] Kawaguchi T., Itou M., Taniguchi E., Sata M. (2014). Exendin-4, aglucagon-like peptide-1 receptor agonist, modulateshepatic fatty acid composition and -Δ5-desaturase index in a murine model of non-alcoholic steatohepatitis. Int. J. Mol. Med..

[50] Rakipovski G., Rolin B., Nøhr J., Klewe I., Frederiksen K.S., Augustin R., Hecksher-Sørensen J., Ingvorsen C., Polex-Wolf J., Knudsen L.B. (2018). The GLP-1 Analogs Liraglutide and Semaglutide Reduce Atherosclerosis in ApoE^−/−^ and LDLr^−/−^ Mice by a Mechanism That Includes Inflammatory Pathways. JACC Basic Transl. Sci..

[51] Kleiner D.E., Brunt E.M., Van Natta M., Behling C., Contos M.J., Cummings O.W., Ferrell L.D., Liu Y.-C., Torbenson M.S., Unalp-Arida A. (2005). Design and validation of a histological scoring system for nonalcoholic fatty liver disease. Hepatology.

[52] Opazo-Ríos L., Soto-Catalán M., Lázaro I., Sala-Vila A., Jiménez-Castilla L., Orejudo M., Moreno J.A., Egido J., Mas-Fontao S. (2022). Meta-Inflammation and De Novo Lipogenesis Markers Are Involved in Metabolic Associated Fatty Liver Disease Progression in BTBR ob/ob Mice. Int. J. Mol. Sci..

