# Supplementary material for: Semaglutide Improves Liver Steatosis and De Novo Lipogenesis Markers in Obese and Type-2-Diabetic Mice with Metabolic-Dysfunction-Associated Steatotic Liver Disease
Source: Int J Mol Sci. 2024 Mar 4;25(5):2961. doi: 10.3390/ijms25052961 (PMC10932050; doi:10.3390/ijms25052961)

Supplementary Table S1. Lipid profile comparison among the three mice strains: BKS Wild Type, BKS db/db, and BKS db/db mice treated with Semaglutide. The values are expressed as median (CI). BKS WT vs BKS db/db: #p < 0.05, ##p < 0.01, ###p < 0.001. BKS db/db vs BKS db/db + Semaglutide: *p < 0.05, **p < 0.01, ***p < 0.001.

Fatty Acid	BKS WT	BKS db/db	BKS db/db + Semaglutide
**C12:0 (µg/mg)**	**0.0104** (0.0079 - 0.0254)	**0.0204** (0.0180 - 0.0319)	**0.0161** (0.0079 - 0.0275)
**C14:0 (µg/mg)**	**0.1063** (0.072 - 0.1491)	**0.5645** (0.5212 - 0.5831) ###	**0.1419** (0.1238 - 0.3771) ***
**C15:0 (µg/mg)**	**0.0189** (0.0153 - 0.0265)	**0.0316** (0.0296 - 0.0512) #	**0.0287** (0.0233 - 0.0545)
**C16:0 (µg/mg)**	**3.920** (2.568 - 5.474)	**18.870** (10.660 - 20.460) ###	**3.659** (2.341 - 6.529) ***
**C16:1n7 (µg/mg)**	**0.8818** (0.4489 - 1.308)	**2.901** (2.414 - 3.194) ###	**0.4227** (0.2859 - 0.8364) ***
**C17:0 (µg/mg)**	**0.0209** (0.0184 - 0.0246)	**0.0502** (0.0478 - 0.0799) ###	**0.0606** (0.0372 - 0.0898)
**C18:0 (µg/mg)**	**0.3219** (0.2868 - 0.3842)	**1.580** (1.424 - 1.774) ###	**0.7114** (0.4873 - 1.240) ***
**C18:1n9cis (µg/mg)**	**6.254** (3.512 - 8.895)	**39.620** (27.730 - 42.490) ###	**5.967** (3.846 - 15.85) ***
**C18:2n6cis (µg/mg)**	**2.083** (1.124 - 3.623)	**4.045** (3.397 - 4.333)	**3.648** (2.656 - 6.045)
**C18:3n3 (µg/mg)**	**0.0448** (0.0343 - 0.0741)	**0.1007** (0.0856 - 0.1116) ##	**0.1575** (0.0987 - 0.1721)
**C20:0 (µg/mg)**	**0.0274** (0.0077 - 0.0355)	**0.0183** (0.0146 - 0.0285)	**0.0285** (0.0113 - 0.0644)
**C20:1n9 (µg/mg)**	**3.920** (2.568 - 5.474)	**18.87** (10.660 - 20.460) ###	**3.659** (2.341 - 6.529) ***
**C20:2n6 (µg/mg)**	**0.0326** (0.0235 - 0.0510)	**0.0502** (0.0437 - 0.1011)	**0.1072** (0.0686 - 0.1336)
**C20:3n6 (µg/mg)**	**0.0868** (0.0560 - 0.1263)	**0.0513** (0.0482 - 0.0847)	**0.1093** (0.0901 - 0.1497) *
**C20:4n6 (µg/mg)**	**0.1651** (0.0775 - 0.2080)	**0.0740** (0.0638 - 0.1124) #	**0.1594** (0.1312 - 0.2436) *
**C20:5n3 (µg/mg)**	**0.0054** (0.0024 - 0.0076)	**0.0040** (0.0029 - 0.0056)	**0.0192** (0.0100 - 0.0370) ***
**C22:1n9 (µg/mg)**	**0.8818** (0.4489 - 1.3080)	**2.9010** (2.4140 - 3.1940) ###	**0.4227** (0.2859 - 0.8364) ***
**C24:0 (µg/mg)**	**0.0104** (0.0079 - 0.0254)	**0.0204** (0.018 - 0.0319)	**0.0161** (0.0079 - 0.0275) **
**C22:6n3 (µg/mg)**	**0.0628** (0.0317 - 0.0879)	**0.0351** (0.0324 - 0.0654)	**0.1239** (0.1040 - 0.2413) ***

Supplementary Figure S1. Experimental design. Leptin receptor-deficient mice (BKS db/db, 12-week-old males) were randomized to untreated and semaglutide-treated (25 μg/kg/week for 2 weeks followed by 100 μg/kg/week for 8 weeks). BKS wild-type mice (BKS WT) littermates served as controls. Mice at 23 weeks-old were sacrificed (euthanized).

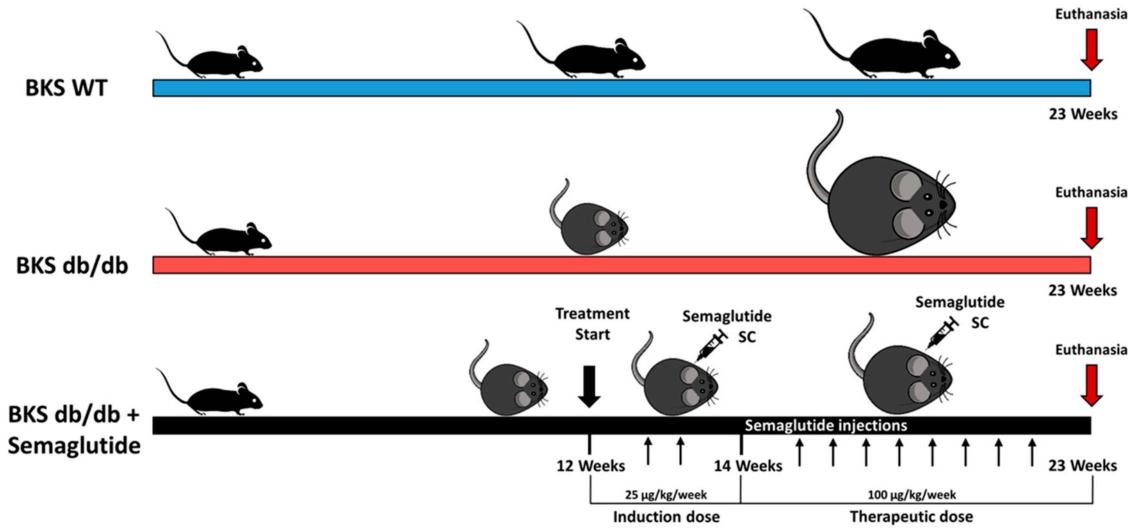

Supplement: Supplementary file 1 [file ijms-25-02961-s001.zip › ijms-2881309-supplementary.pdf]
